# A Comparative Study of the Common Protozoan Parasites of *Clarias gariepinus* from the Wild and Cultured Environments in Benue State, Nigeria

**DOI:** 10.1155/2011/916489

**Published:** 2011-10-18

**Authors:** S. Omeji, S. G. Solomon, E. S. Idoga

**Affiliations:** ^1^Department of Fisheries and Aquaculture, University of Agriculture, PMB 2373 Makurdi, Benue State, Nigeria; ^2^Department of Vetrinary Medicine, Ahmadu Bello University, Zaria, Nigeria

## Abstract

A total of one hundred and twenty *Clarias gariepinus* comprising 30 dead and 30 live fishes were examined for protozoan parasites infestation, sixty each from the wild and a pond (cultured environment) over a period of six months. *Ichthyophthirius multifiliis* was the most common protozoan parasites found in *C. gariepinus* from the wild (River Benue) and cultured (pond) environments. These protozoan parasites constitute 37.08% of the total parasites encountered for fishes in the pond and 42.51% of fishes in the wild. Among the body parts of the sampled fishes from the pond, the gills had the highest parasite load (38.86%). Also, the gills had the highest parasite load (40.54%) among the body parts of the fishes sampled from the wild. Fishes not infested with any protozoan parasites from the pond constituted 36.70% of the total fish sampled. On the other hand, fishes not infested with any protozoan parasites from the wild constituted 31.65% of the total fish sampled. Female fishes had more protozoan parasites than the male fishes. Bigger fishes of total length (25–48 cm) had more parasite load than the smaller ones (19–24 cm). Also, fishes between 150–750 g had more parasite load than the smaller ones of less than 150 g. Protozoan parasite load of fish from the cultured environment (pond) did not differ significantly (*P* < 0.05) from those from River Benue (wild).

## 1. Introduction

Fish is important to human populace in trade and economy; it is of importance in the diet of different countries especially in the tropics and subtropics where malnutrition is a major problem [1]. As the human population inevitably increases, the demand for fish as source of protein also grows. In recent times, there has been tremendous increase in the development of fish farming and culture attributable to the increased need for affordable animal protein especially in the tropics [2], therefore, catfishes of the family Clariidae are increasingly being used for freshwater aquaculture in Africa owing to several favourable cultural characteristics [3]. Parasitic infection and diseases are some of the factors hindering high productivity in fish farming [4, 5]. 

According to Klinger and Francis-Floyd [6] protozoa are a vast assemblage of eukaryotic organisms and that most of the commonly encountered fish parasites are protozoa, which with practice, are the easiest to identify and easiest to control. In general protozoa are one of the major sectors of fish parasites that have been long neglected because of its inherent difficulty in studying compared to other larger parasites. Among protozoa, ecto- and endoparasitic protozoa occupy a very important sector as one of the hazardous threats to fish health. These parasites attack the fish, causing massive destruction of skin and gill epithelium. Even moderate infection of these organisms on small fish may prove a fatal disease, since the infection may cause the fish to stop feeding [7].

Some fish parasites would develop in humans if the fish is eaten raw, but none would be harmful if the fish is thoroughly cooked. All reports of people being infested with fish parasites were because of ingestion of raw fish or insufficiently cooked fish [8]. Most fish especially in the wild population are likely to be infested with parasites, but in the great majority of cases, no significant harm to the host may be ensued or identified; thus, there are only few reports of parasites causing mortality or serious damage to the fish populations, but this may be largely because such effects go unnoticed [9]. Fishermen or consumers often observe parasites in wild fish only when they are so obvious as to lead to rejection of fish [10]. In culture fish population, on the other hand, parasites often cause serious outbreak of disease. The presence of dense populations of fish kept in particular environmental conditions may favour certain parasites so that the parasite population increases to a very high level. According to Roberts et al. [11], parasites are the most diverse and common pathogens the aquaculturist may likely encounter, and parasitic diseases are very common in fish all over the world and are of particular importance in the tropics.

Parasite of fish can either be external or internal. Parasitic infections often give an indication of the quality of water, since parasites generally increase in abundance and diversity in more polluted waters [12, 13]. Parasites are capable of causing harm to the fish host notwithstanding the sp., either through injury to the tissues or organs in the process of burrowing or consuming food or the removal of digested food in the gut of the fish as well as the secretion of proteolytic enzymes.

Fish parasites result in economic losses not only mortality, but also from treatment expenses, growth reduction during and after outbreak of disease and this militate against expansion of aquaculture. Protozoan parasites cause serious losses in fishponds and wild in Nigeria, and their lesions render the fish unmarketable. Fish carrying protozoa parasites are capable of passing on the infective disease to man after its consumption. 

Protozoa are common tropical freshwater fish parasites that affect public health and cause losses to fishes, hence its choice for this study.

## 2. Materials and Methods

The study took place in Makurdi the capital of Benue State, Nigeria, located at longitude 7°43′ N and latitude 8°32′ E. The town is divided into the north and south bank by the River Benue. River Benue exists year round though the water volume fluctuates with season. The river overflows its banks during the rainy season (May–October) but decreases drastically in volume leaving tiny island in the middle of the river during the dry season (November–April). The river contains several sp. of freshwater fishes of different families. 

One hundred and twenty *Clarias gariepinus* (60 each from the wild and a pond), comprising 30 live and 30 dead fishes of different sizes were bought from local fishermen along the course of River Benue (The wild), and from Jab-Bella farm (pond) both in Wadata, Benue state, Nigeria. Five samples each from both the wild and pond were collected fortnightly for a period of six (6) months, June–November 2008. The fishes were identified using the field guide to Nigerian freshwater fishes by Babatunde and Aminu [14]. The total and standard lengths of each fish were measured in centimeters (cm) using meter rule, while the weight of each of the fishes was taken in grams (g) using an electronic meter balance. The sexes of the fishes were also determined after examination of their papillae. 

External examination of each of the fish for parasites was carried out using the technique of Emere and Egbe [15] on the gills, fins and skin. The skin, gill and fins of each of the fish were also examined for ectoparasites using hand lens. The fish samples were also filleted using scalpel blade. The tissue was placed on a Petri-dish and 3 mL of 0.9% saline solution were added and stirred using a mounted pin. Some drops of the mixed solution were collected using dropper, placed on a slide, and then covered with a cover slip after which, observation on a light binocular microscope was made. Later, the gills of each of the fish were dissected using a dissecting kit, each of the gill was placed in 10 mL of normal saline in Petri-dish, later removed and then place on a slide on which 1-2 drops of saline solution were added and observed on a binocular microscope. The stomach and the intestine of each of the fish were cut opened, and contents washed into the Petri-dish containing the saline solution. The lining of the gut lumen was also scrapped out and placed in the saline solution. One to two drops of the preparation were placed on slide covered with slips and observed using a light binocular microscope for endoparasites. Ectoparasitic data were collected on the gills, fins, and skins of the fish, while the endoparasitic data were collected on the stomach and intestine of the fish using the techniques of Emere and Egbe [15]. 

 The parasites were identified by making their sketches as observed on the binocular microscope and compared with the pictorial guide on fish parasites by Pouder et al. [16]. The parasites observed on the binocular microscope were counted and recorded. Two-way analysis of variance was use to determine significant differences in sex, source, and status of the specimens. The ANOVA was carried out using GENSTAT Discovery Edition from Lawes Agricultural Trust Rothamsted.

## 3. Results

Results of the 30 dead and 30 live *C. gariepinus *from the cultured environment (pond) used for the study are as shown in Table 1. Out of the 30 dead *C. gariepinus *used, 14 (46.7%) were not infested by any protozoan parasites, while 16 (53.3%) of them were infested with protozoan parasites and were observed to harbour a total number of 86 protozoan parasites.

Among the parasites found on the parts of the sampled fishes, *I. multifiliis* was the most abundant 28 (32.56%), followed by *Ichthyobodo *sp. 18 (20.93%), *Trichodina *sp. 18 (20.93%), *Cryptobia iubilans* 15 (17.44%), and lastly *Chilodonella *sp. 7 (8.14%). It was observed that the gill has the highest load of protozoan parasites (34%) followed by the skin (29%), while the intestine, fin, and stomach accounted for 10%, 8%, and 5%, respectively. Whereas, out of the 30 live *C. gariepinus *used, 8 (26.7%) were not infested by any protozoan parasites, while 22 (73.3%) of them were infested by protozoan parasites and were observed to harbour a total number of 125 protozoan parasites. *I. multifiliis* were found on the gill and skin, *Ichthyobodo *sp. appeared on the gill, *Trichodina *sp. were found on the skin and fin *Chilodonella *sp. were found on the skin while *Cryptobia iubilans* were found in the stomach and intestine. *I. multifiliis* was the most abundant 52 (41.60%), followed by *Cryptobia iubilans* 30 (24.00%), *Ichthyobodo *sp. 23 (18.40%), *Trichodina* sp. 17 (13.60%), and lastly *Chilodonella *sp. 3 (2.40%). It was also observed that the gill has the highest load of protozoan parasites (48%) followed by the skin (36%), while the intestine, fin, and stomach accounted for 16%, 14%, and 11%, respectively. It was observed that live *C. gariepinus *from the pond has more protozoan parasites than dead *C. gariepinus *from the same pond.

Results of the 30 dead and 30 live *C. gariepinus *from the River Benue (wild) used for the study are as shown in Table 2. Out of the 30 dead *C. gariepinus *used, 12 (40%) were not infested by any protozoan parasites, while 18 (60%) of them were infested by protozoan parasites and were observed to harbour a total number of 91 protozoan parasites.

From the above (Table 2), *I. multifiliis* were found on the gill and skin and was the most abundant 39 (42.86%) followed by *Cryptobia. iubilans*, which were found in the stomach and intestine 27 (29.67%), *Ichthyobodo *sp. on the gill 16 (17.58%), *Trichodina* sp. on the fin 5 (5.49%), and lastly *Chilodonella *sp. on the skin 4 (3.40%). It was observed that the gill has the highest load of protozoan parasites (36%), followed by the skin (23%), while the fin, stomach, and intestine accounted for 5%, 11%, and 16%, respectively. Nevertheless, out of the 30 live *C. gariepinus *from the wild, used for the study, 7 (23.3%) were not infested by any protozoan parasites, while 23 (76.7%) of them were infested by protozoan parasites and were observed to harbour a total number of 131 protozoan parasites. *I. multifiliis* were found on the gill and skin and was the most abundant 55 (41.98%) followed by *Cryptobia iubilans* 28 (21.37%), which were found in the stomach and intestine, *Ichthyobodo* sp. on the gill 30 (22.90%), *Trichodina* sp. on the skin and fin 14 (10.69%), and lastly *Chilodonella* sp. on the skin 4 (3.05%). It was observed that the gill has the highest load of protozoan parasites (54%) followed by the skin (39%), while the intestine, stomach, and fin accounted for 17%, 11%, and 10%, respectively. It was also observed that live *C. gariepinus *from the wild has more protozoan parasites than dead *C. gariepinus *from the same wild.

**Figure 1:**
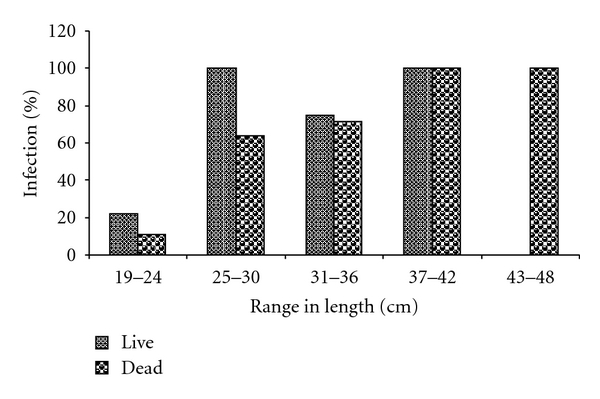
Size distribution and percentage parasite infection in dead and live* C. gariepinus *from the cultured environment (pond).

**Figure 2:**
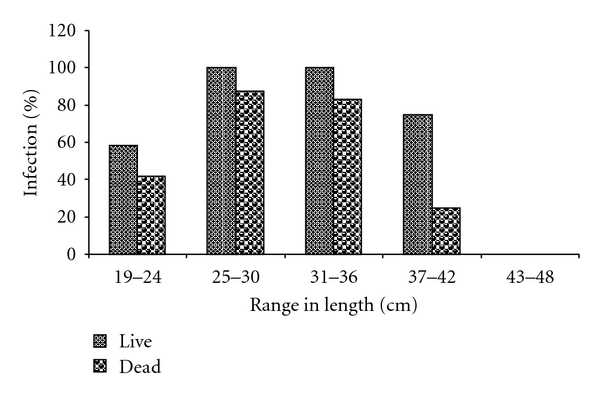
Size distribution and percentage parasite infection in dead and live *C. gariepinus *from River Benue (wild).

Result of the size distribution and percentage parasite infection in dead and live *C. gariepinus* from the cultured environment (pond) is as shown in Figure 1, while Figure 2 shows the result of size distribution and percentage parasite infection in dead and live *C. gariepinus* from the River Benue (wild). From these figures, it was observed that bigger fishes of total length between 25 cm–48 cm were more infected than smaller fishes (total length between 19 cm–24 cm) from both sources. 

**Figure 3:**
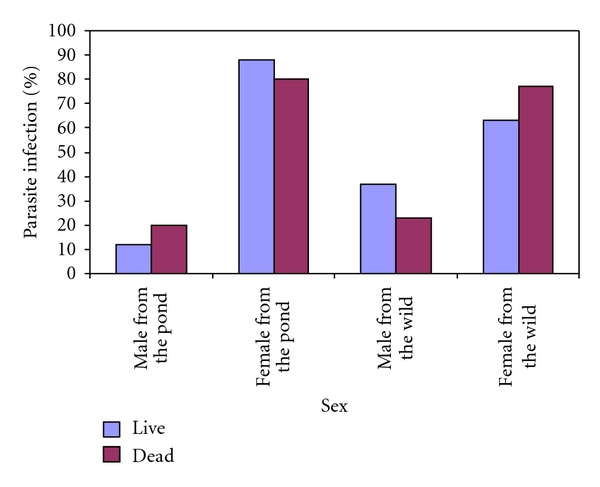
Sex and percentage parasite infection in dead and live *C. gariepinus* from the cultured environment (pond) and River Benue.

Results of sex and percentage parasite infection in dead and live *C. gariepinus* from the cultured environment (pond) and River Benue are as shown in Figure 3. The results show that the dead female *C. gariepinus* from the pond had a greater rate of infection (80.23%) than the dead male (19.76%), and the live female *C. gariepinus* had a greater rate of infection (88.00%) than the live male (12.00%). In addition, the dead female *C. gariepinus* from the River Benue had a greater rate of infection (77.17%) than the dead male (22.82%), and the live female had a greater rate of infection (63.36%) than the live male (36.64%). 

Results of the percentage parasites load on the different body parts of both dead male and female and that of live male and female *C. gariepinus* with respect to the weight of the fishes from the cultured environment (pond) are as shown in Tables 3 and 4. While Tables 5 and 6 show the results of the percentage parasites load on the different body parts of both dead male and female and that of live male and female *C. gariepinus* with respect to the weight of the fishes from River Benue. The results show that fishes with bigger weight (150–750 g) had more parasites than smaller fishes with less than 150 g.

The correlation matrix for the total number of parasites found on* C. gariepinus *by size, from both sources is as shown in Table 7.

From the above, there was a high correlation (0.724) between the total length (TL) and total number of parasites (TNP) for dead *C. gariepinus* collected from the cultured environment (pond). In contrast, there was a low correlation (0.434) between the total length (TL) and total number of parasites (TNP) for dead *C. gariepinus *caught from River Benue. In addition, there was a high correlation (0.669) between the total number of parasites (TNP) and weight (WT) for dead *C. gariepinus* collected from the cultured environment (pond) but a low correlation (0.213) between the total number of parasites (TNP) and weight for dead *C. gariepinus *caught from River Benue. Also, in the cultured environment (pond), a high correlation value (0.830) was recorded between the total length (TL) and total number of parasites (TNP) for live *C. gariepinus *but a low correlation value (0.403) was recorded between the total length (TL) and total number of parasites (TNP) for live *C. gariepinus* caught from River Benue. In addition, there was a high correlation (0.758) between the total number of parasites (TNP) and weight (WT) for live *C. gariepinus *collected from the cultured environment (pond) and 0.510 between the total number of parasites (TNP) and weight (WT) for live *C. gariepinus *caught from River Benue. 

## 4. Damaging Effects of Parasites on the Sampled Fishes

**Figure 4:**
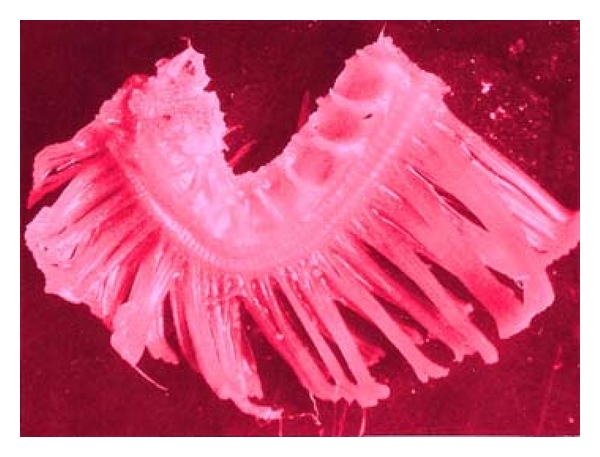
Gill of *Clarias gariepinus *showing excess mucus production.

**Figure 5:**
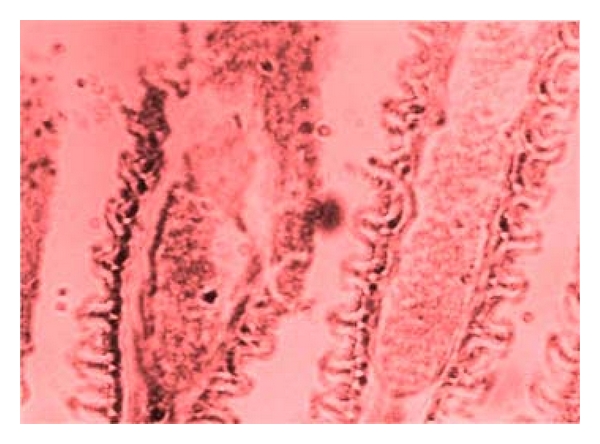
Gill of *Clarias gariepinus *showing thickened gill epithelium.

**Figure 6:**
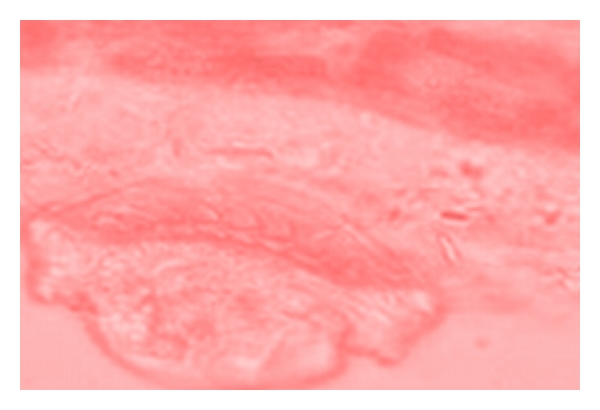
*Trichodina* sp. on the skin of *C. gariepinus*.

**Figure 7:**
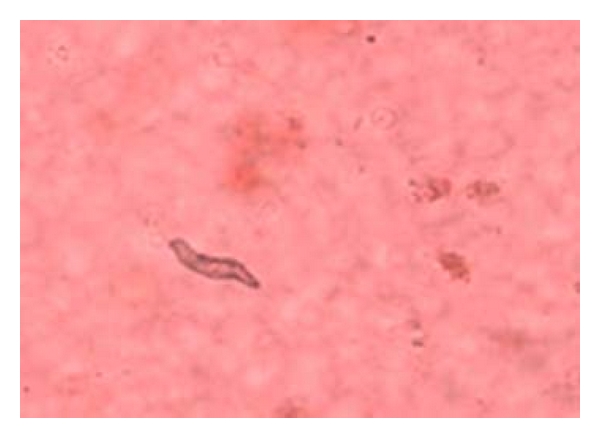
*Cryptobia iubilans* on the stomach epithelium of *C. gariepinus*.

**Figure 8:**
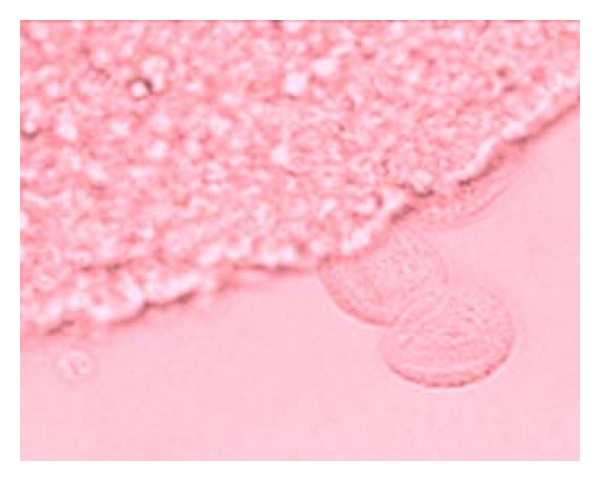
*Chilodonella *sp. on the skin of *C. gariepinus*.

Several damages were observed to have been caused by the parasites found on the body parts of the sampled fishes. Erosion of the epithelium on the skin and thickening of the gills (as seen in Figure 5), as well as excess mucus secretion on the gills of the sampled fishes (Figure 4), was caused by* I. multifiliis*. This caused restriction of the oxygen flow from the water to the blood in the gills of infected fishes. The respiratory folds of the gills, the lamellae, also become deformed, reducing the transfer of oxygen. The shear numbers of *I. multifiliis* covering the gills also could cause mechanical blockage of oxygen transfer. These conditions combine to stress the fish by hindering respiration. The epithelial layer of the gill may separate and cause loss of electrolytes, nutrients, and fluids from the fish, making it difficult for the fish to regulate the water concentration in its body. Secondary bacteria and fungi also invade the fish more easily, while it is impaired from the *I. multifiliis* infection. Death in infected fishes resulted from asphyxiation. Excess mucus production and removal of the skin epithelium was caused by *Trichodina *sp., as seen on Figure 6. This resulted in sluggish movement, loss of appetite, emaciation, loss of condition with larger head and darker skin than normal. Some infected fish showed pale skin patches and more slimy skin. Excess mucus formation on the skin was also observed in the sampled fishes infected by *Chilodonella *sp. (Figure 8). This made the skin to appear slimy and exhibited cloudiness, and showed evidence of irritation as it tried to “scratch” off the organisms by rubbing against the walls of the fish pond. The fish also exhibited lethargy. *Cryptobia iubilans* in the stomach of sampled fishes (Figure 7) caused erosion of the stomach epithelium. Infected fish showed inappetance, decreased activity, and stayed isolated from other fish. 

## 5. Discussion

Different kinds of protozoan parasites were observed to be present in different locations in *C. gariepinus. I. multifiliis* occurred on the gill and skin where chronic infections of the fishes were observed, *Trichodina *sp. were found on the skin and fin, *Ichthyobodo *sp. and *Chilodonella * sp. were found on the skin, while *Cryptobia iubilans* were found in the stomach and intestine. Emere and Egbe [15] and Hines and Spira [17, 18] had reported the infection of the skin, fin, and gills of fish by these protozoan parasites.

 The present study revealed that *Cryptobia iubilans* affected only the intestine and stomach of the fish studied, and in addition, these parasites were more in the intestine than the stomach, but Somerville [19] in his work reported that a large number of *Cryptobia *protozoan were found on the external surface of cultured rainbow trout in USA. The occurrence of *Cryptobia iubilans* in the intestine than the stomach either might be due to the presence of digested food present there or due to the greater surface area presented by the intestine [20]. Smith [21] reported that most protozoan parasites inhabit the intestine because of their general feeding habits. Reduced number of the protozoan parasites in the stomach might be due to the movement of the stomach muscle and acid (HCL) nature of the stomach. Adebanjo [20] observed that the acid nature of the stomach might inhibit the parasites there. E. E. Noble and G. R. Noble [22] also reported that protozoan parasites prefer a certain pH medium.

Gills were also observed to harbour the highest number of protozoan parasites. This could be because the gills are the center of filter feeding and are the sites of gaseous exchange. This observation agrees with the reported works of Emere and Egbe [15], who reported highest load of protozoan parasites in the gill of *Synodontis clarias* and Nyaku et al. [23] who reported highest load of protozoan parasites in the gills of *Auchenoglanis ocidentalis*,* Oreochromis niloticus*, and *Bagrus bayad* in River Benue. Investigation by Roger and Gainer [24] and Chakroff [25] had shown the gills to be infested by different protozoan parasites. According to Somerville [19], the sieving ability of the gill rakers may help to trap some organisms, and this could be attributed to the presence of the protozoan parasites there.

*I. multifiliis *caused erosion of the epithelium and thickening of the gills, this could be attributed to inflammatory processes which occurred during infection with this parasitic ciliate as described by Sigh et al. [26]. Infection with *Trichodina* sp. caused removal of the epithelium and excess mucus production so that the fin and gills of infected fishes were covered with a thick layer of mucus. This agrees with the reported work of Obiekezie and Ekanem [3]. 

The heavy load of parasites on the gills relative to other parts of the body impaired the gills from functioning well as an organ of respiration, hence death could result. This agrees with the reported work of Borg [27], Omoniyi et al. [28], and Rahman et al. [29]. 

The female fishes had the greatest rate of protozoan parasite infestations than the male counterparts. This might be connected to the physiological state of the females. Most gravid females could have had reduced resistance to infection by parasites. In addition, their increased rate of food intake to meet their food requirements for the development of their egg might have exposed them to more contact with the parasites, which subsequently increased their chance of being infected. Emere and Egbe [15], Adebanjo [20], and Holden and Reed [30] had made similar observation.

 Bigger fishes were observed to have higher rate of protozoan parasites than the smaller ones. This might be because the bigger ones cover wider areas in search of food. As a result, they take in more food than the smaller ones, and this exposed them more to infection by parasites. In addition, they are omnivorous and feed on anything that comes their ways. Emere and Egbe [15] and Holden and Reed [30] had made similar observation in *S. clarias*.

 Protozoan parasite load of fish from the pond did not differ significantly from those from the wild. However, the significantly higher load of parasites in the live fish as compared to the dead could be attributed to parasite migration as a result of dead of the host (fish) which occurred soon after they died, prior to the examination as described by Klinger and Francis-Floyd [6].

## Figures and Tables

**Table 1:** Protozoa parasites and their locations in dead and live *C. gariepinus* from the cultured environment (pond).

Protozoa parasites	Number of fish infected by each protozoa parasite		Location of parasites	Percentage parasite infection per location		Parasite load on each location		Percentage parasite sp. on fish	
	Dead	Live		Dead	Live	Dead	Live	Dead	Live
*Ichthyobodo *sp.	6	7	gill	39.53	38.40	18	23	20.93	18.40
*I. multifiliis*	6	6	gill			16	25	32.56	41.60
*I. multifiliis*	5	9	skin			12	27		
*Chilodonella* sp.	3	2	skin	33.72	28.80	7	3	8.14	2.40
*Trichodina* sp.	4	3	skin			10	6	20.93	13.60
*Trichodina* sp.	3	6	fin	9.30	8.80	8	11		
*Cryptobia iubilans*	2	4	stomach	5.81	11.20	5	14	17.44	24.00
*Cryptobia iubilans*	4	5	intestine	11.63	12.80	10	16		

**Table 2:** Protozoa parasites and their locations in dead and live *C. gariepinus* from river benue (wild).

Protozoa parasites	Number of fish infected by each protozoa parasite		Location of parasites	Percentage parasite infection per location		Parasite load on each location		Percentage parasite sp. on fish	
	Dead	Live		Dead	Live	Dead	Live	Dead	Live
*Ichthyobodo sp*	7	7	gill	39.56	41.22	16	30	21.98	18.32
*I. multifiliis*	6	8	gill			20	24	38.46	46.56
*I. multifiliis*	6	10	skin			19	31		
*Chilodonella* sp.	3	2	skin	25.27	29.77	4	4	3.40	3.05
*Trichodina* sp.	0	0	skin			0	4	5.49	10.69
*Trichodina* sp.	4	6	fin	5.49	7.63	5	10		
*Cryptobia iubilans*	4	6	stomach	12.09	8.40	11	11	29.69	21.37
*Cryptobia iubilans*	5	5	intestine	17.58	12.98	16	17		

**Table 3:** Percentage parasite load on/in the body parts of dead male and female *C. gariepinus* from the pond.

Weight of Fish	percentage (%) parasite load									
	Gill		Skin		Fin		Stomach		Intestine	
	Male	Female	Male	Female	Male	Female	Male	Female	Male	Female
<150 g	60	53	0	33	0	13	40	0	0	0
150–250 g	25	38	33	48	0	10	25	0	17	3
250–350 g	0	29	0	29	0	0	0	0	0	43
350–450 g	0	0	0	0	0	0	0	0	0	0
450–550 g	0	25	0	25	0	0	0	0	0	50
550–650 g	0	50	0	20	0	30	0	0	0	0
650–750 g	0	0	0	0	0	0	0	0	0	0

**Table 4:** Percentage parasite load on/in the body parts of live male and female *C. gariepinus* from the pond.

Weight of Fish	percentage (%) parasite load									
	Gill		Skin		Fin		Stomach		Intestine	
	Male	Female	Male	Female	Male	Female	Male	Female	Male	Female
<150 g	20	39	53	39	7	7	20	13	0	0
150–250 g	0	64	0	0	0	7	0	0	0	26
250–350 g	0	26	0	23	0	14	0	11	0	26
350–450 g	0	38	0	22	0	14	0	8	0	18
450–550 g	0	35	0	35	0	0	0	17	0	13
550–650 g	0	0	0	0	0	0	0	0	0	0
650–750 g	0	0	0	0	0	0	0	0	0	0

**Table 5:** Percentage parasite load on/in the body parts of dead male and female *C. gariepinus* from the wild.

Weight of fish	percentage (%) parasite load									
	Gill		Skin		Fin		Stomach		Intestine	
	Male	Female	Male	Female	Male	Female	Male	Female	Male	Female
<150 g	21	48	50	13	7	17	0	17	21	4
150–250 g	33	45	67	16	0	0	0	8	0	32
250–350 g	0	0	0	0	0	0	0	0	0	0
350–450 g	0	30	0	30	0	0	0	40	0	0
450–550 g	0	0	0	0	0	0	0	0	0	0
550–650 g	0	0	0	0	0	0	0	0	0	0
650–750 g	0	0	0	0	0	0	0	0	0	0

**Table 6:** Percentage parasite load on/in the body parts of live male and female *C. gariepinus* from the wild.

Weight of fish	percentage (%) parasite load									
	Gill		Skin		Fin		Stomach		Intestine	
	Male	Female	Male	Female	Male	Female	Male	Female	Male	Female
<150 g	25	65	19	15	50	3	6	12	0	6
150–250 g	33	50	33	44	13	0	0	0	20	6
250–350 g	0	42	50	25	0	0	50	0	0	33
350–450 g	0	44	0	33	0	0	0	22	0	0
450–550 g	25	25	42	42	0	0	0	0	33	33
550–650 g	0	0	0	0	0	0	0	0	0	0
650–750 g	0	0	0	0	0	0	0	0	0	0

**Table 7:** Correlation matrix for total number of parasites found on* C. gariepinus *by size.

	Dead *C. gariepinus* from the cultured environment (pond)			Live *C. gariepinus* from the cultured environment (pond)		
	TL	TNP	WT	TL	TNP	WT
TL	1.00			1.00		
TNP	0.724	1.00		0.830	1.00	
WT	0.864	0.669	1.00	0.889	0.758	1.00

	Dead *C. gariepinus* from river benue			Live *C. gariepinus* from river benue		
	TL	TNP	WT	TL	TNP	WT

TL	1.00			1.00		
TNP	0.434	1.00		0.403	1.00	
WT	0.893	0.213	1.00	0.916	0.510	1.00

TL: total length, TNP: total number of parasites, and WT: weight of fish.
